# Genome-wide association study identifies loci and candidate genes for non-idiopathic pulmonary hypertension in Eastern Chinese Han population

**DOI:** 10.1186/s12890-018-0719-0

**Published:** 2018-10-05

**Authors:** Caiyong Yin, Kai Li, Yanfang Yu, Huijie Huang, Youjia Yu, Zhongqun Wang, Jinchuan Yan, Yan Pu, Zheng Li, Ding Li, Peng Chen, Feng Chen

**Affiliations:** 1 Department of Forensic Medicine, Nanjing Medical University, Nanjing, Jiangsu 211166 People’s Republic of China; 2 MOE Key Laboratory of Contemporary Anthropology, Department of Anthropology and Human Genetics, School of Life Sciences, Fudan University, Shanghai, 200438 People’s Republic of China; 3 Department of Cardiology, Affiliated Hospital of Jiangsu University, Zhenjiang, Jiangsu 212001 People’s Republic of China; 4 School of Medicine, Southeast University, Nanjing, Jiangsu 210009 People’s Republic of China; 5 Key Laboratory of Targeted Intervention of Cardiovascular Disease, Collaborative Innovation Center for Cardiovascular Disease Translational Medicine, Nanjing Medical University, Nanjing, Jiangsu 211166 People’s Republic of China

**Keywords:** Pulmonary hypertension, Nox3, Tbx4, GWAS

## Abstract

**Background:**

Pulmonary hypertension (PH) is a rare disease characterized by proliferation and occlusion of small pulmonary arterioles, which has been associated with a high mortality rate. The pathogenesis of PH is complex and incompletely understood, which includes both genetic and environmental factors that alter vascular structure and function.

**Methods:**

Thus we aimed to reveal the potential genetic etiology of PH by targeting 143 tag SNPs of 14 candidate genes. Totally 208 individuals from Chinese Han population were enrolled in the present study, including 109 non-idiopathic PH patients and 99 healthy controls.

**Results:**

The data revealed that 2 SNPs were associated with PH overall susceptibility at *p* < 3×10^− 4^ after Bonferroni correction. The top hit was rs6557421 (*p* = 4.5×10^− 9^), located within *Nox3* gene on chromosome 6. Another SNP rs3744439 located in *Tbx4* gene, also showed evidence of association with PH susceptibility (*p* = 1.2×10^− 6^). The distribution of genotype frequencies of rs6557421 and rs3744439 have dramatic differences between PH patients and controls. Individuals with rs6557421 TT genotype had a 10.72-fold/14.20-fold increased risk to develop PH when compared with GG or GG/GT carriers in codominant or recessive model, respectively (TT versus GG: 95%CI = 4.79–24.00; TT versus GG/GT: 95%CI = 6.65–30.33). As for rs3744439, AG genotype only occurred in healthy controls but has not been observed in PH patients. We further validated the result by using 26 different populations from five regions around the globe, including African (AFR), American (AMR), East Asian (EAS), European (EUR), and South Asian (SAS). In consistent with the present case-control study’s results, significantly different genotype frequencies of the observed SNPs existed between PH patients and healthy individuals from all over the world.

**Conclusions:**

The results suggested that rs6557421 variant in *Nox3* and rs3744439 variant in *Tbx4* might have potential effect on individual susceptibility to pulmonary hypertension, which could lead to therapeutic or diagnosis approaches in PH.

## Background

Pulmonary hypertension (PH) is a rare disease characterized by proliferation and occlusion of small pulmonary arterioles, leading to progressive elevation of pulmonary artery pressure, pulmonary vascular resistance, and right ventricular failure [1]. There are 3 subtypes of PH according to the National Organization for Rare Disorders (NORD), including heritable pulmonary hypertension (HPH), idiopathic pulmonary arterial hypertension (IPH) and associated pulmonary hypertension (APH) [2]. More than half of the PH patients are non-idiopathic PH, which are also known as secondary PH. During the present study, we focused on the genetic susceptibility of non-idiopathic PH. Revealing the genetic etiology of non-idiopathic PH would facilitate diagnosis and development of novel therapies in the future. During the present study, we reviewed and screened several candidate genes that are potentially or directly associated with PH occurrence (Table 1).

**Table 1:** Summarized 14 genes that were analyzed in the present study

Gene name	The (potential) relationship between the studied genes and PH occurrence.
*Solute carrier family 4 member 4* (*SLC4A4, also known as NBCe1*)	*SLC4A4* encodes NBCe1 which is the first eukaryotic Na^+^ − coupled transporter. The transporter is a member of the anion exchange family belonging to the second largest secondary carrier superfamily (APC). A previous GWAS study have revealed that SLC4A4 was hypertension susceptibility gene [12].
*NADPH oxidase 3 (Nox3)*	Nox3 is a member of the NADPH oxidase (Nox) family of oxidant-generating enzymes. After its original cloning and detection in fetal tissues and inner ear, studies remain limited and its function mostly limited to gravity perception. However, several studies have also reported the physiologic role of Nox3 induction in lungs and lung endothelial cells [7, 13].
*Interleukin 6 (IL6)*	Interleukin 6 (IL6) is a pleiotropic cytokine with a wide range of biologic function on hematopoiesis, inflammation, immune regulation and oncogenesis. The increasing levels of IL-6 in lung and serum are associated with PH [3].
*Transcription factor EC (TFEC)*	Transcription factor EC (TFEC) most likely acts as a transcriptional repressor in heterodimers with other microphthalmia-TFE (MiT) family members. The expression of murine TFEC is restricted to macrophages (mTFEC). mTFEC as a macrophage-specific transcription factor plays a critical role in macrophage-specific gene regulation [3]. Considering the activities of macrophages in PH development, TFEC might have potential connection with PH.
*Caveolin-1*	Caveolin-1, which is a major protein constituent of caveolae, interacts with a variety of signaling molecules implicated in PH. Disruption and progressive loss of endothelial caveolin-1 with reciprocal activation of proliferative pathways occur before the onset of PH, and the rescue of caveolin-1 inhibits proliferative pathways and attenuates PH [4].
*NADPH oxidase 4 (Nox4)*	NADPH oxidase 4 is the major NADPH oxidase homolog expressed in human PASMCs and its expression both at the mRNA and protein level is significantly increased in lungs from patients with PH, which suggests a correlation between Nox4 and the onset of PH [5, 6]. Additionally, Nox4 acts as a primary source of ROS, contributes to the proliferation and remodeling of PH [7, 8].
*Oxidized low density lipoprotein receptor 1 (OLR-1)*	Oxidized low density lipoprotein receptor 1 (OLR-1), the receptor for oxidized low-density lipoprotein, is expressed in endothelial cells, macrophages or smooth muscle cells. Recent studies have shown that OLR-1 is involved in the lung inflammation and injury [3].
*Thrombospondin 1 (THBS1)*	Thrombospondin 1 (THBS1) is a kind of matricellular protein, which is a secreted molecular that has both extracellular matrix and cell surface receptor. In animals, pulmonary THBS1 is upregulated rapidly following hypoxic challenge. Additionally, clinical studies of plasma THBS1 and mutations of THBS1 in PH have been reported [4].
*ATPase phospholipid transporting 8B4 (ATP8B4)*	ATPase phospholipid transporting 8B4 (ATP8B4) activity is important in ATP biosynthesis and phospholipid transport via a variety of potential mechanisms. A recent whole-exome sequencing research identified that ATP8B4 gene is strongly associated with the risk of development of PH [5].
*NADPH oxidase 5 (Nox5)*	NADPH oxidase 5 (Nox5) is the last NOX family member to be identified. Caveolin-1 binds directly to Nox5 and suppresses the activity of Nox5. Although the direct connection between Nox5 and PH has not been reported, dysregulation of caveolin-1 has been documented with PH states.
*T-box4 (Tbx4)*	T-box4 (Tbx4) is a transcription factor in the T-box gene family, which is expressed in variety of organs including mesenchyme of the lung and trachea. Tbx4 has been shown to be involved in lung growth and branching. The function mutations in Tbx4 have been previously reported to be associated with PH [3].
*Chromobox 7 (CBX7)*	Chromobox 7 (CBX7) is one of the five mammalian orthologues of Drosophila Polycomb. CBX7 was recognized as the main orthologue of Drosophila Polycomb implicated in maintaining the self-renewal of embryonic stem cells. Until now, litter is known about the potential correlation of CBX7 to PH.
*Cytochrome b-245 beta chain (CYBB)*	The *Cytochrome b-245 beta chain (CYBB)* gene encodes membrane protein Nox2, which plays a crucial role in stromal interaction molecular 1 (STIM1) activation and in the control of the cytosolic oscillations that drive murine pulmonary microvascular endothelial cells (MPMVECs) activation [4]. Our recent research has also revealed the role of STIM1 during the acute lung intoxication [5].
*Galectin-3 (Gal-3)*	Galectin-3 (Gal-3) is a β-galactoside binding lectin that regulates multiple pathways. Our recent study has revealed the effectiveness of genetic and pharmacological strategies targeting Gal-3 in halting the progression of PH remodeling and development of experimental PH [1].

In summary, these genes were shown to be associated or potentially related with PH development though the molecular mechanism had not been well understood. To further clarify the association and reveal the candidate functional variants, we conducted the limited scale genome wide association study. One hundred forty-three tag SNPs were screened from the sequences between the upstream and downstream 2Kb of the 14 studied genes. The screening population data are from Chinese Han population in Beijing (CHB) of the Hapmap Project (NCBI build 36, dbSNPb126) and the candidate SNPs were screened by using Haploview software.

## Methods

### Samples

Totally 208 individuals were enrolled in the present study, including 109 non-idiopathic PH patients and 99 healthy controls. Patients were consecutively recruited from the Affiliated Hospital of Jiangsu University between May 2014 and July 2016. Clinical information was obtained from medical records, including gender, age, drink, smoke and coronary artery disease (CAD) history and so on. Baseline profiles of the studied population were summarized in Table 2. None of the patients have connective tissue diseases, HIV infection, portal hypertension, congenital heart diseases or thyroid dysfunction. The control subjects were collected from healthy volunteers who visited the Sir Run Run Hospital Nanjing Medical University for medical examination during the same period. The study was performed with the approval of the ethics committee of the Nanjing Medical University and the informed consent was obtained from each participant.

**Table 2:** Demographic and clinical characteristics of the subjects

	Patients	Controls
Sex		
Male	50 (0.46)	63 (0.62)
Female	59 (0.54)	36 (0.38)
Age (y)	74.07 ±12.86	37.26±9.18
Systolic PAP (mmHg)	60.67 ± 12.73	
Drink		
Yes	6 (0.06)	
No	103 (0.94)	
Smoke		
Yes	16 (0.15)	
No	93 (0.85)	
Coronary heart disease		
Yes	66 (0.60)	
No	43 (0.40)	
Heart failure (HF)		
Yes	17 (0.15)	
No	92 (0.84)	
Echocardiogram		
LA (mm)	48.62 ± 10.58	
LvIDd (mm)	52.18 ± 9.77	
IVSD (mm)	10.60 ± 4.80	
LVEF (%)	57.85 ± 15.40	

*PAP* pulmonary artery pressure, *LA* atrial diameter, *LvIDd* left ventricular end diastolic diameter, *IVST* interventricular septal thickness, *LVEF* left ventricular ejection fraction

### Experimental design and Genotyping

**Fig. 1:**
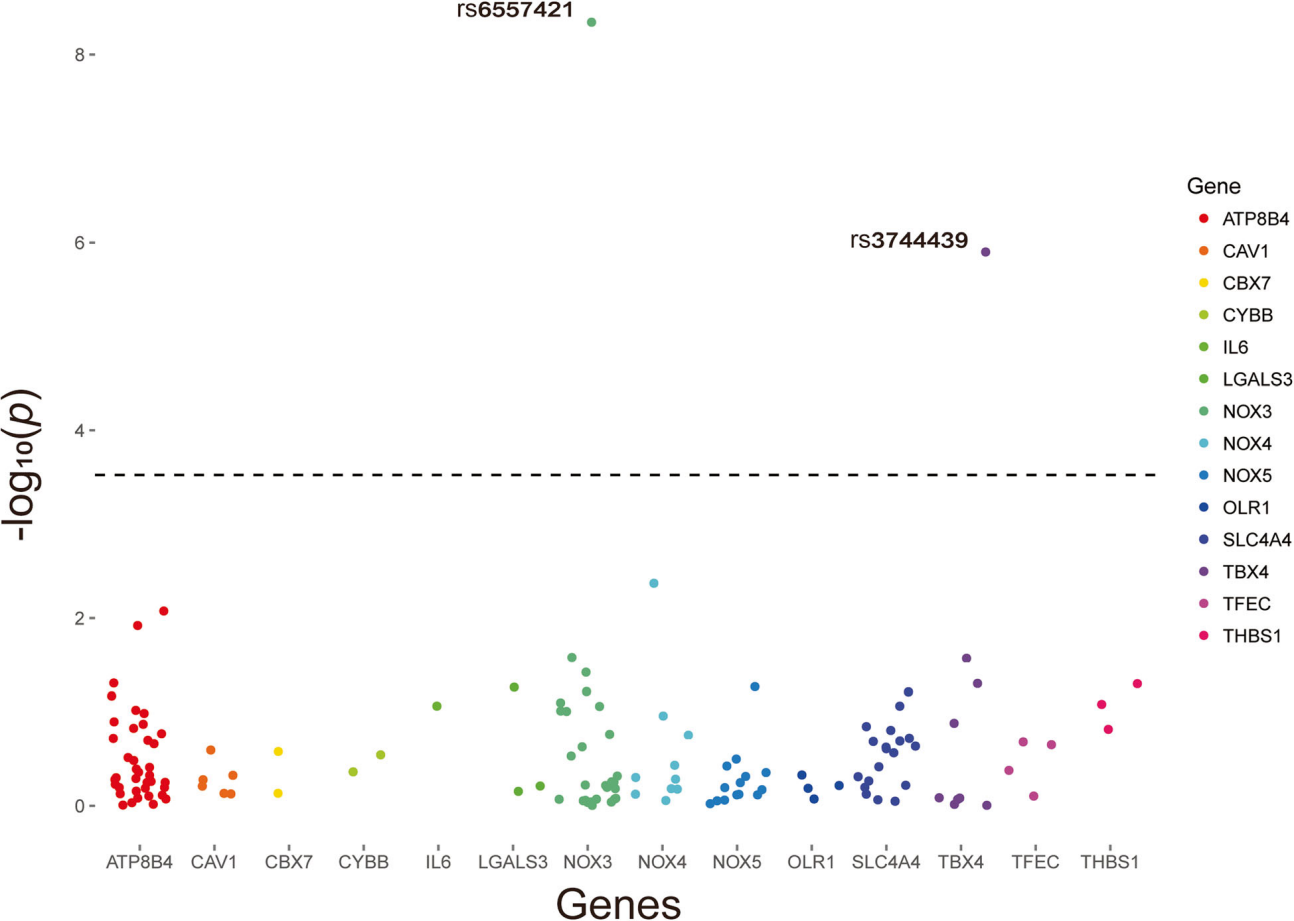
Manhattan plot for SNPs in the different genes (*n* = 143)

Genomic DNA was extracted from 200 μl EDTA-anticoagulated peripheral blood using a commercial extraction kit (Tiangen Biotech Corporation, Beijing, China) according to the instruction manual. The 143 tag SNPs in 109 PH patients were firstly genotyped by using Illumina X-10 platform, and the sequencing was done by commercial company (Decode Genomics BioTech Co., Ltd, Nanjing, China). The genotyping results were then compared with healthy individuals from Southern Han Chinese (CHS) population of 1000G database. And 2 SNPs emerged at a significant level (*P* < 3×10^-4^) (Fig. 1). To confirm the finding, we further genotyped 2 SNPs in 99 healthy individuals from Nanjing, Jiangsu by performing fluorescent PCR and Ligase detection reaction (LDR). The primers were designed by using Primer 3 online software version 0.4.0 (http://frodo.wi.mit.edu/) and Oligo software version 6.3.1 (Molecular Biology Insights, USA). The SNPs were amplified in a final volume of 20 μl that contained 50 ng of DNA, 2 μl 1×buffer, 0.6 μl 3 mM Mg2+, 2 μl 2 mM dNTP, 0.2 μl Taq DNA polymerase (1 unit/μl) and appropriate concentrations of primers. The PCR reaction was subjected to an initial denaturation at 95 °C for 2 min, followed by 40 cycles of amplification consisting of denaturation at 94 °C for 30 s, annealing at 53 °C for 90 s, extension at 65 °C for 30 s, followed by final extension at 65 °C for 10 mim. The LDR was carried out on the PRISM 3730 DNA analyzer (Thermo Fisher Scientific, USA). The LDR were performed in a total volume of 10 μl that contained 4 μl PCR production, 1 μl 1×buffer, 1 μl probe mix, 0.05 μl Taq DNA polymerase (2 unit/μl). The LDR condition was 95 °C for 2 mim, followed by 40 cycles of 15 s at 94 °C and 25 s at 50 °C. The SNPs were further genotyped by using Genemapper.

### Statistical analysis

All data were analyzed by using SPSS 19 (SPSS Inc., Chicago, IL). Genotype frequencies of SNPs were obtained by directed computing. Genotypic association analyses in a case-control pattern assuming codominant, dominant, recessive and overdominant genetic models were performed using SNPstats [3]. Odds ratio (OR) and respective 95% confidence interval were reported to evaluate the effects of any differences between allele and genotype frequencies.

## Results

### Candidate SNP selection

Totally 143 SNPs were successfully genotyped in 109 PH patients. After comparing the genotyping results with the data generated from 105 individuals from Southern Han Chinese population in 1000G database, the results demonstrated that 2 SNPs were associated with PH with overall susceptibility at *p* < 3×10^− 4^ after Bonferroni adjustment. The related analysis was shown in Fig. 1. The top hit was rs6557421 (*p* = 4.5×10^− 9^), which is located in *Nox3* gene on chromosome 6. Another SNP rs3744439 located in *Tbx4* gene, also showed evidence of association with PH susceptibility (*p* = 1.2×10^− 6^).

**Fig. 2:**
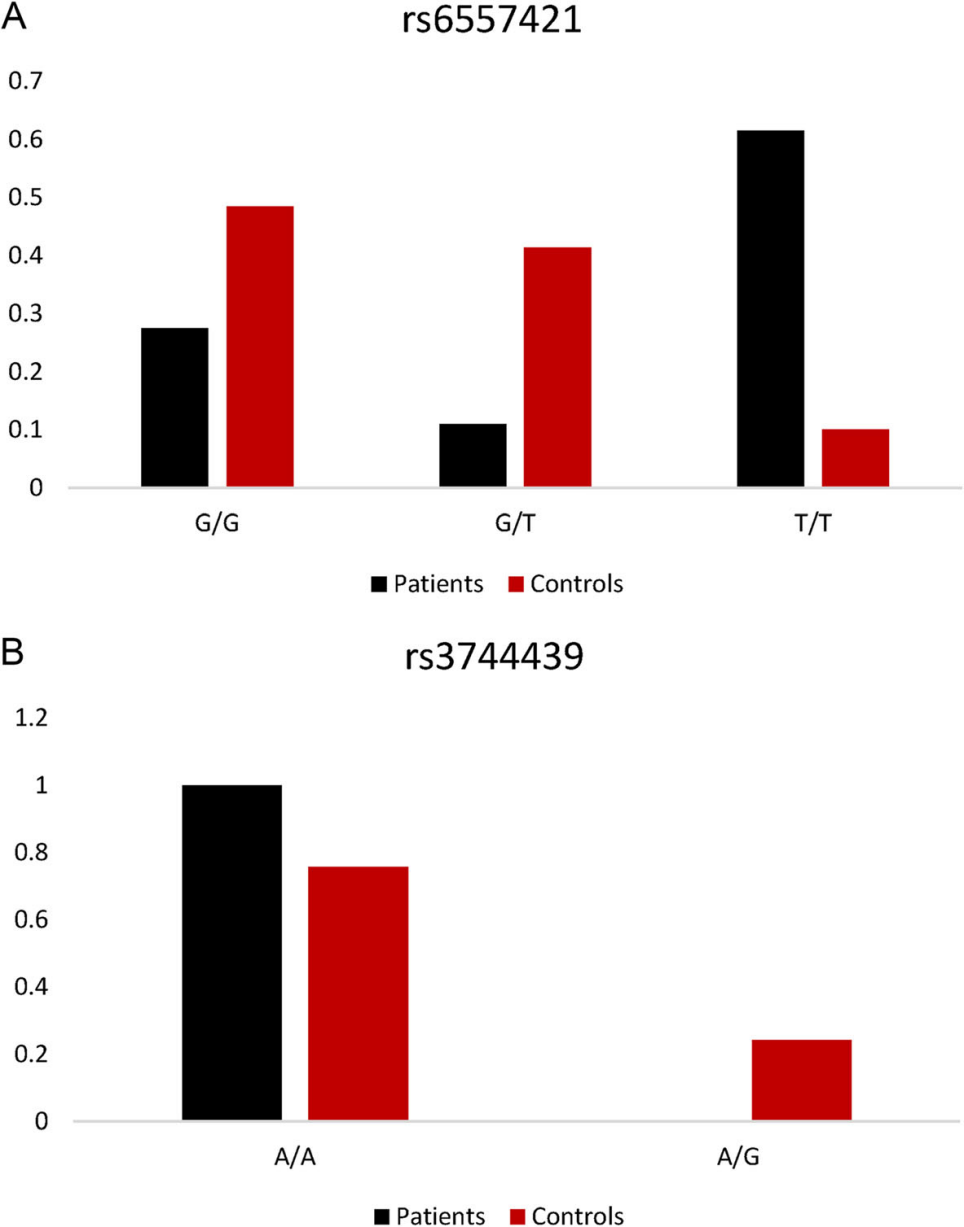
The distribution of genotype frequencies of *Nox3* rs6557421 (**a**) and *Tbx4* rs3744439 (**b**) among PH patients and healthy controls

We further genotyped the 2 SNPs in 99 healthy control subjects. The distribution of genotype frequencies of rs6557421 and rs3744439 was illustrated in Fig. 2. Dramatic differences existed between PH patients and controls. As shown in Table 3, individuals with rs6557421 TT genotype had a 10.72-fold/14.20-fold increased risk to develop PH when compared with GG or GG/GT carriers in codominant or recessive model, respectively (TT versus GG: 95%CI = 4.79–24.00; TT versus GG/GT: 95%CI = 6.65–30.33). In dominant model, significantly increased PH susceptibility was associated with GT/TT genotypes compared with GG genotype (OR = 2.48, 95%CI = 1.39–4.41). Furthermore, dramatically decreased PH risk was associated with GT genotype when compared with GG/TT genotypes in an overdominant model.

**Table 3:** Genotype frequencies of rs6557421 in *Nox3* gene among PH patients and controls

Genetic model	Genotype	Patients	Controls	Logistic regression		Logistic regression (adjusted)	
		*N* = 109 (%)	*N* = 99 (%)	OR (95%CI)	*P*-value	OR^c^ (95%CI)	*P*-value
Codominant	GG	30 (27.5)	48 (48.5)	1.00		1.00	
	GT	12 (11)	41 (41.4)	0.47 (0.21–1.03)	**< 0.0001**	0.22 (0.03–1.66)	**0.0001**
	TT	67 (61.5)	10 (10.1)	**10.72 (4.79–24.00)**		**9.66 (1.82–51.28)**	
Dominant	GG	30 (27.5)	48 (48.5)	1		1.00	
	GT/TT	79 (72.5)	51 (51.5)	**2.48 (1.39–4.41)**	**0.0018**	2.02 (0.53–7.74)	0.3
Recessive	GG/GT	42 (38.5)	89 (89.9)	1.00		1.00	
	TT	67 (61.5)	10 (10.1)	**14.20 (6.65–30.33)**	**< 0.0001**	**17.29 (3.60–83.02)**	**0.0001**
Overdominant	GG/TT	97 (89)	58 (58.6)	1.00		1.00	
	GT	12 (11)	41 (41.4)	**0.18 (0.09–0.36)**	**< 0.0001**	**0.08 (0.01–0.48)**	**0.0013**

Bold-faced values indicate a significant difference at the 5% level; ^c^adjusted by sex and age

As shown in Fig. 2b and Table 4, rs3744439 AG genotype only occurred in healthy controls which had not been observed in PH patients.

**Table 4:** Genoetype frequencies of rs3744439 in *Tbx4* gene among *PH* patients and controls

Genetic model	Genotype	Patients	Controls	Logistic regression		Logistic regression (adjusted)	
		*N* = 109 (%)	*N* = 99 (%)	OR (95%CI)	*P*-value	OR^c^ (95%CI)	*P*-value
	AA	109 (100)	75 (75.8)	1.00		1.00	
	AG	0 (0)	24 (24.2)	0.00 (0.00-NA)	**< 0.0001**	0.00 (0.00-NA)	**0.0013**

Bold-faced values indicate a significant difference at the 5% level; ^c^adjusted by sex and age

**Fig. 3:**
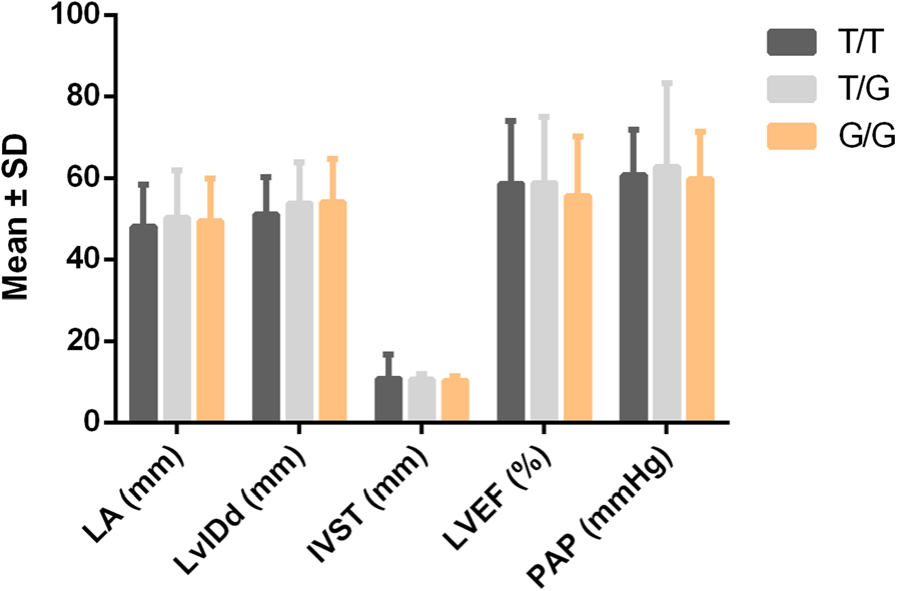
The distribution of patients’ left atrial diameter (LA), left ventricular end diastolic diameter (LvIDd), interventricular septal thickness (IVST), left ventricular ejection fraction (LVEF) and systolic pulmonary artery pressure (PAP) among different genotypes of *Nox3* rs6557421

Stratification analysis was also performed, and PH patients were divided into three groups according to the *Nox3* rs6557421 genotypes. The distribution of patients’ left atrial diameter (LA), left ventricular end diastolic diameter (LvIDd), interventricular septal thickness (IVST), left ventricular ejection fraction (LVEF) and systolic pulmonary artery pressure (PAP) were compared between each groups (Fig. 3). No significant differences were observed among different genotyping groups.

### Comparison between different populations around globe

**Fig. 4:**
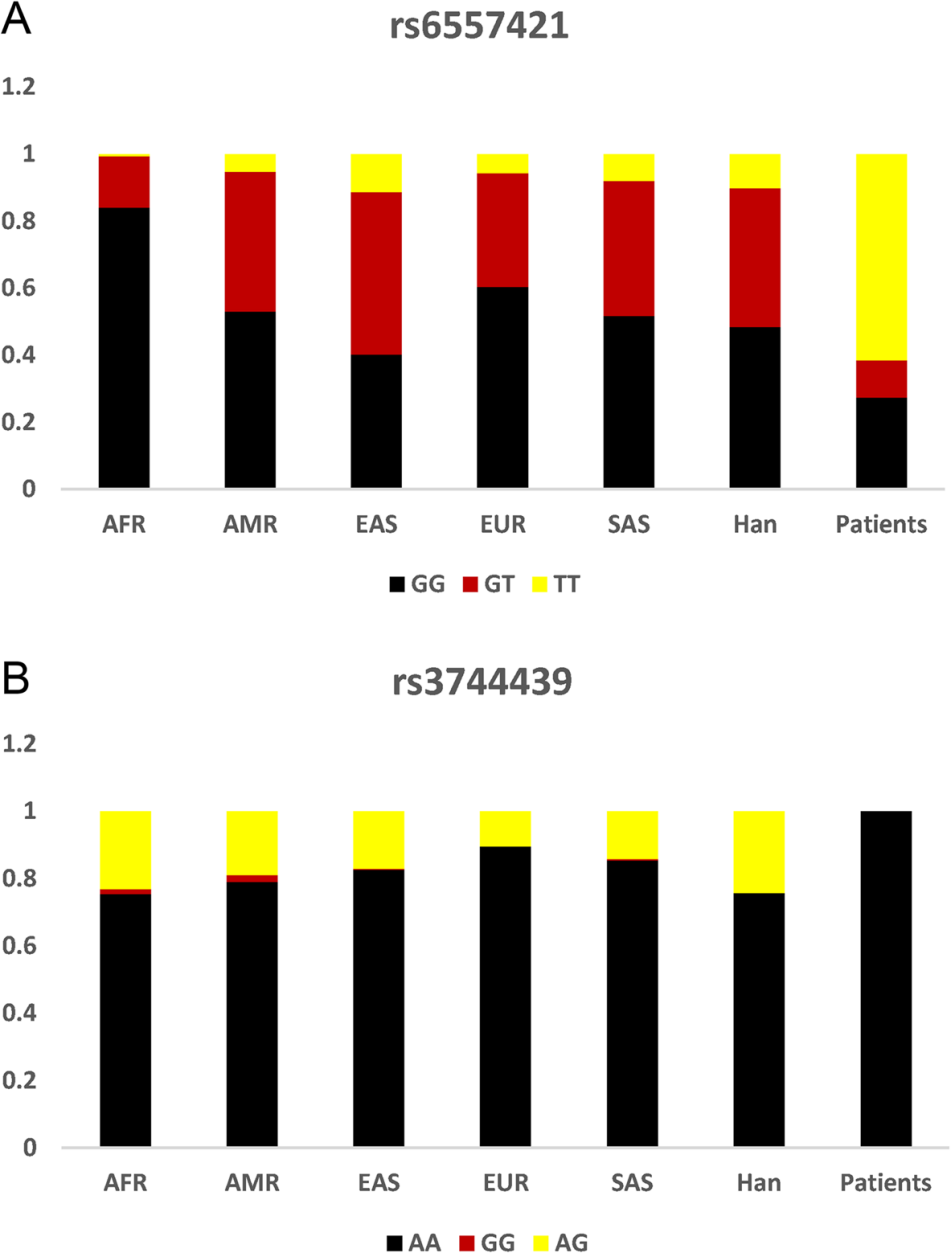
The distribution of genotype frequencies of *Nox3* rs6557421 (**a**) and *Tbx4* rs3744439 (**b**) among the studied population and 26 populations from 1000G

We further validated the observation by using 26 different populations from five regions around the globe, including African (AFR), American (AMR), East Asian (EAS), European (EUR), and South Asian (SAS). As illustrated in Fig. 4a and b, the distribution of rs6557421 and rs3744439 genotypes’ frequencies of control group from Chinese Han population was quite similar to which from Asian populations. In consistent to the present case-control study’s results, significantly different genotype frequencies existed between PH Patients and healthy individuals from all over the world (Fig. 4a and b).

## Discussion

Among NADPH oxidases, Nox3 seems to have the most restricted expression pattern in the mammalian organism. Nox3 is specifically localized to the inner ear. Thus the previous studies mostly focused on the unique function of Nox3 involving in the biogenesis of otoconia/otolith [4]. However, few studies have revealed the potential critical role of Nox3 in the pathophysiological process of PH development. Nox3 is the predominant source of palmitate-induced ROS generation [5]. Nox3 activation during ischemia-reperfusion injury may contribute to the development of primary graft dysfunction after lung transplantation [6]. Upregulated Nox3 leads to increased oxidant generation and elastolytic activity, resulting in increased oxidant injury and death in mice and lung endothelial cells [7]. Treatment of *Tlr*^−/−^ mice or endothelial cells with *Nox3* siRNA or chemical NADPH inhibitors could reverse the phenotype [7]. Overall, the studies identified that Nox3 could be a potential therapeutic target in the lungs.

The present case-control study revealed that a *Nox3* tagSNP rs6557421 had a significantly association with the PH susceptibility. When random individuals carry rs6557421 TT genotype, they have nearly 10-fold increased risk to develop PH when compared with GG or GG/GT carriers. Moreover, GT genotype carriers have a significantly decreased PH risk compared with GG/TT genotype carriers. To further investigate the racial disparities, we enrolled 26 populations from 1000G to make a comparison. All the populations from different continents possessed relatively low frequency of TT genotype (AFR = 0.006, AMR = 0.052, EAS = 0.113, EUR = 0.056, SAS = 0.08, Han in present = 0.101). On the contrary, the proportion of TT genotype carriers in PH patients reached 0.615. The data indicated that TT genotype might be associated with increased PH in different races.

Mutations in *Tbx4* have been proved to be associated with PH onset [8]. Tbx4 shows high specific expression in lung fibroblasts and broadly regulates fibroblast-related pathway that partly contributes to super-enhancer-mediated transcriptional programs [9]. Decreased Tbx4 in the pulmonary mesenchyme during fetal lung development may lead to the decrease or arrest of airway branching, thus contributing to PH [10]. Tbx4 was also regarded as a mesenchymal transcription factor that drove myofibroblasts accumulation and the development of lung fibrosis [11].

Our research revealed that *Tbx4* rs3744439 had a significant association with PH risk. The frequencies of AG genotype in present control Chinese Han population and other 26 populations around the globe are all over than 0.10 (Han in present = 0.24, AFR = 0.23, AMR = 0.19, EAS = 0.17, EUR = 0.10, SAS = 0.14). Interestingly, during the PH patients, AG and GG genotype frequencies were 0. The data suggested that AG genotype could be a protective factor for the PH development.

The present study has an obvious limitation. As noted in the Methods section that the ages of patients and controls are significantly different. The controls are younger, and they might develop PH in the future. Considering the relative low incidence of PH, we believe the results are reliable. To further validate the results, healthy independent individuals from 1000G database were also included. And the results were consistent.

## Conclusions

In summary, it is biologically plausible that rs6557421 variant in *Nox3* and rs3744439 variant in *Tbx4* may have  potential effects on individual susceptibility to pulmonary hypertension, which could lead to therapeutic or diagnosis approaches in PH.

## Abbreviations

ATP8B4: ATPase phospholipid transporting 8B4
CHX7: Chromobox 7
CYBB: Cytochrome b-245 beta chain
IL6: Interleukin 6
NORD: National Organization for Rare Disorders
Nox3: NADPH oxidase 3
Nox4: NADPH oxidase 4
Nox5: NADPH oxidase 5
OLR-1: Oxidized low density lipoprotein receptor 1
PH: Pulmonary hypertension
SLC4A4: Solute carrier family 4 member 4
Tbx4: T-box4
TFEC: Transcription factor EC
THBS1: Thrombospondin 1
